# Oral Manifestations of Juvenile Behçet Syndrome: A Case Report and Mini-Review of the Literature

**DOI:** 10.7759/cureus.114367

**Published:** 2026-08-11

**Authors:** Safaa El Omari, Oumaima Barhoud, Samira Elarabi, Sana Bensouda

**Affiliations:** 1 Department of Pediatric Dentistry, Faculty of Dental Medicine, Hassan II University of Casablanca, Ibn Rochd University Hospital Center, Casablanca, MAR; 2 Department of Pediatric Dentistry, Faculty of Dental Medicine, Hassan II University of Casablanca, Casablanca, MAR

**Keywords:** hla-b51, juvenile behçet syndrome, oral ulceration, pediatric behçet's disease, pediatric dentistry

## Abstract

Behçet’s disease is a chronic, relapsing, multisystem inflammatory vasculitis characterized by a broad spectrum of mucocutaneous and systemic manifestations. Oral ulcerations are often the earliest and most frequent clinical feature, playing a central role in diagnosis, particularly in pediatric patients, in whom the disease remains uncommon and its diagnosis is frequently delayed. We report the case of a boy aged 11 years and 6 months with juvenile Behçet syndrome who presented with recurrent oral aphthous ulcerations, including an 8-mm ulcer on the left lateral border of the tongue and a 5-mm ulcer on the external surface of the upper lip, along with poor oral hygiene and generalized gingival inflammation. The patient had a history of arthralgia and was receiving colchicine (1 mg/day). Oral management consisted of reinforcement of oral hygiene and dietary measures, full-mouth scaling, and topical treatment with corticosteroids and propolis-based therapy. At the 10-day follow-up, complete healing of the initial oral lesions and marked improvement in gingival inflammation were observed, although new aphthous ulcers developed at different intraoral sites, consistent with the relapsing nature of the disease. This case highlights the importance of early recognition of oral manifestations in Pediatric Behçet’s Disease (PEDBD) and emphasizes the key role of pediatric dentists in facilitating diagnosis, multidisciplinary management, and long-term follow-up. Appropriate oral care may contribute to improved symptom control, enhanced quality of life, and the prevention of oral complications.

## Introduction

Behçet syndrome (BS), also known as Behçet’s disease, is a chronic, relapsing, multisystem inflammatory vasculitis characterized by recurrent episodes of exacerbation and remission. First described in 1937 by the Turkish dermatologist Hulusi Behçet, the disease is classically defined by a triad of recurrent oral aphthous ulcers, genital ulcerations, and ocular involvement [1]. However, BS is a systemic disorder with a broad spectrum of clinical manifestations that may affect the musculoskeletal, neurological, gastrointestinal, and vascular systems [2].

Pediatric-onset BS accounts for approximately 15-20% of all cases and often presents with heterogeneous or incomplete clinical manifestations, contributing to delayed diagnosis [2,3]. BS exhibits a characteristic geographic distribution along the historic Silk Road, extending from the Mediterranean basin to East Asia, with its prevalence reaching up to 370 cases per 100,000 inhabitants in some regions along this route, whereas it remains relatively uncommon in Western countries [4]. The etiology and pathogenesis of BS remain incompletely understood and are thought to result from a complex interaction between genetic susceptibility and environmental factors, including microbial triggers and oral microbiome dysbiosis [2, 5, 6].

Recurrent oral ulceration is often the earliest and most frequent clinical manifestation and may precede the onset of systemic involvement, highlighting its diagnostic importance, particularly in children [2, 7]. The diagnosis remains primarily clinical and is supported by established classification criteria, including those of the International Study Group (ISG), the International Criteria for Behçet’s Disease (ICBD), and the Pediatric Behçet’s Disease (PEDBD) criteria. However, these criteria have limitations in children, in whom incomplete or atypical presentations may delay diagnosis [2,5].

The management of BS aims to control inflammation, reduce disease activity, and prevent irreversible organ damage through a multidisciplinary approach. Mucocutaneous lesions are primarily managed with topical corticosteroids and colchicine, whereas systemic corticosteroids and immunosuppressive agents are used for severe or refractory disease [3,4]. Given that oral ulceration is often the earliest and most frequent manifestation, pediatric dentists play a crucial role in the early recognition of the disease and appropriate referral for multidisciplinary care [8, 9].

Although recurrent oral ulceration is the most common and often the earliest manifestation of BS, diagnosis in children is frequently delayed because of the initially incomplete clinical presentation and the gradual development of systemic manifestations. In this context, dentists, particularly pediatric dentists, play a key role in recognizing suggestive oral signs and facilitating timely referral for specialist evaluation, thereby helping to reduce diagnostic delay. However, the literature on pediatric BS originates predominantly from medical specialties and focuses mainly on systemic manifestations and their medical management, whereas the dental perspective remains underrepresented. This case report describes the oral management of a child with BS treated at the Department of Pediatric Dentistry, Casablanca Dental Consultation and Treatment Center (CCTD), Faculty of Dental Medicine, Casablanca, Morocco. Beyond the clinical description, it highlights the value of structured dental follow-up in monitoring recurrent oral lesions, maintaining oral health, reinforcing oral hygiene practices, and preventing local complications. It also emphasizes the importance of integrating dental care into a multidisciplinary approach to optimize overall patient management and improve the quality of life of affected children.

## Case presentation

A Moroccan boy aged 11 years and 6 months with a previously established diagnosis of Behçet syndrome was referred by his treating physician to the Department of Pediatric Dentistry at the CCTD, Casablanca, for the evaluation and management of oral lesions. According to the medical history and a review of the patient’s records, recurrent oral ulcerations and joint involvement first appeared at the age of 2 years. The diagnosis was established by the pediatric rheumatology team at the age of 8 years, after the occurrence of genital ulceration. The patient has since been receiving colchicine (1 mg/day) and remains under regular pediatric rheumatology follow-up for the early detection of potential ocular and other systemic manifestations (Table 1).

**Table 1:** Clinical timeline of the present case. CCTD: Center for Dental Consultations and Treatments; mg/day: Milligrams per day.

Age/time	Clinical event
2 years	Onset of recurrent oral ulcerations and joint involvement.
2-8 years	Continued recurrent oral ulcerations and joint involvement without a definitive diagnosis.
8 years	Development of genital ulceration, diagnosis of Behçet syndrome, and initiation of colchicine therapy (1 mg/day).
11 years and 6 months	Initial dental examination at the Department of Pediatric Dentistry, CCTD, Casablanca, for the evaluation and management of oral manifestations.
10 days after the initial examination	Healing of the initial lesions, with the emergence of new aphthous ulcers at different intraoral sites.
6-month follow-up	Regular visits every 3-4 weeks to monitor oral manifestations and provide preventive dental care. The patient turned 12 years old during this period.

The patient had no significant medical history apart from the manifestations of BS. The family history was negative for BS; however, a history of psoriasis was reported among relatives. According to the patient’s history, recurrent oral aphthous ulcers occurred approximately two to three times per month, with new lesions frequently appearing shortly after the healing of previous ulcers. Each episode persisted for approximately three to four days before resolving, either following topical treatment or spontaneously. The patient reported moderate discomfort associated with these lesions, which were described as bothersome during active episodes.

**Figure 1:**
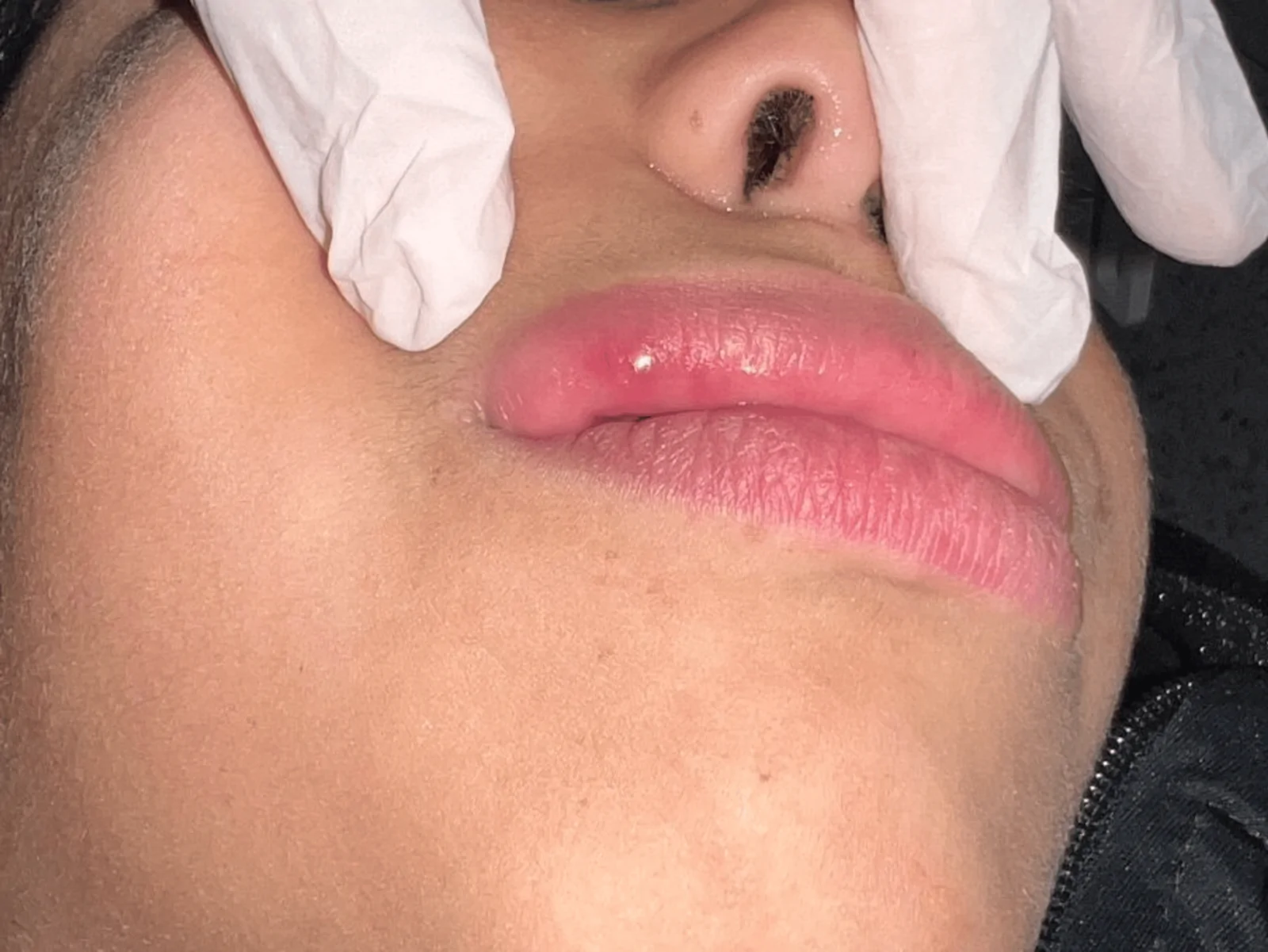
Extraoral view showing a 5-mm aphthous ulcer on the external surface of the upper lip.

**Figure 2:**
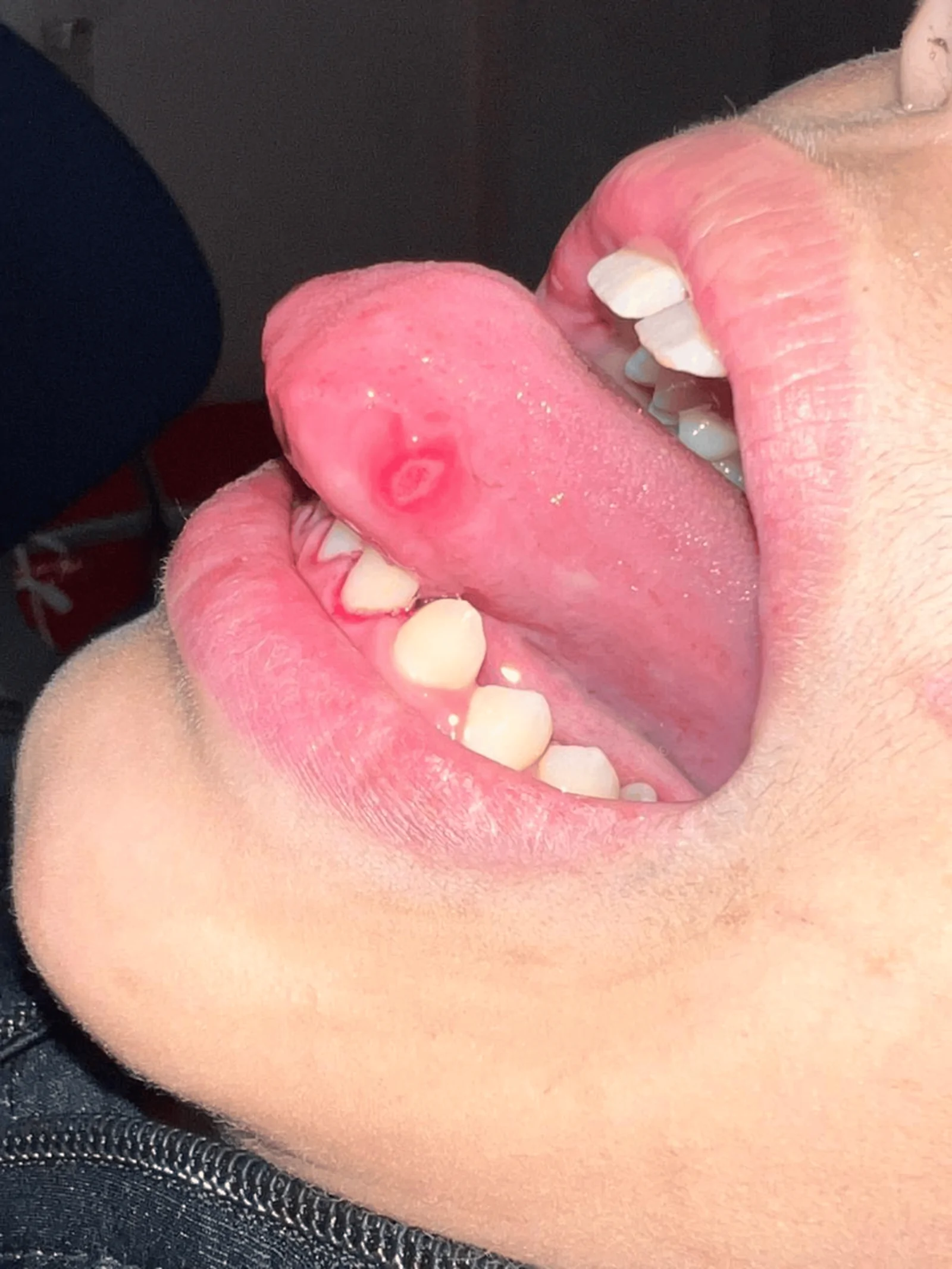
Intraoral view showing an 8-mm aphthous ulcer on the left lateral border of the tongue.

**Figure 3:**
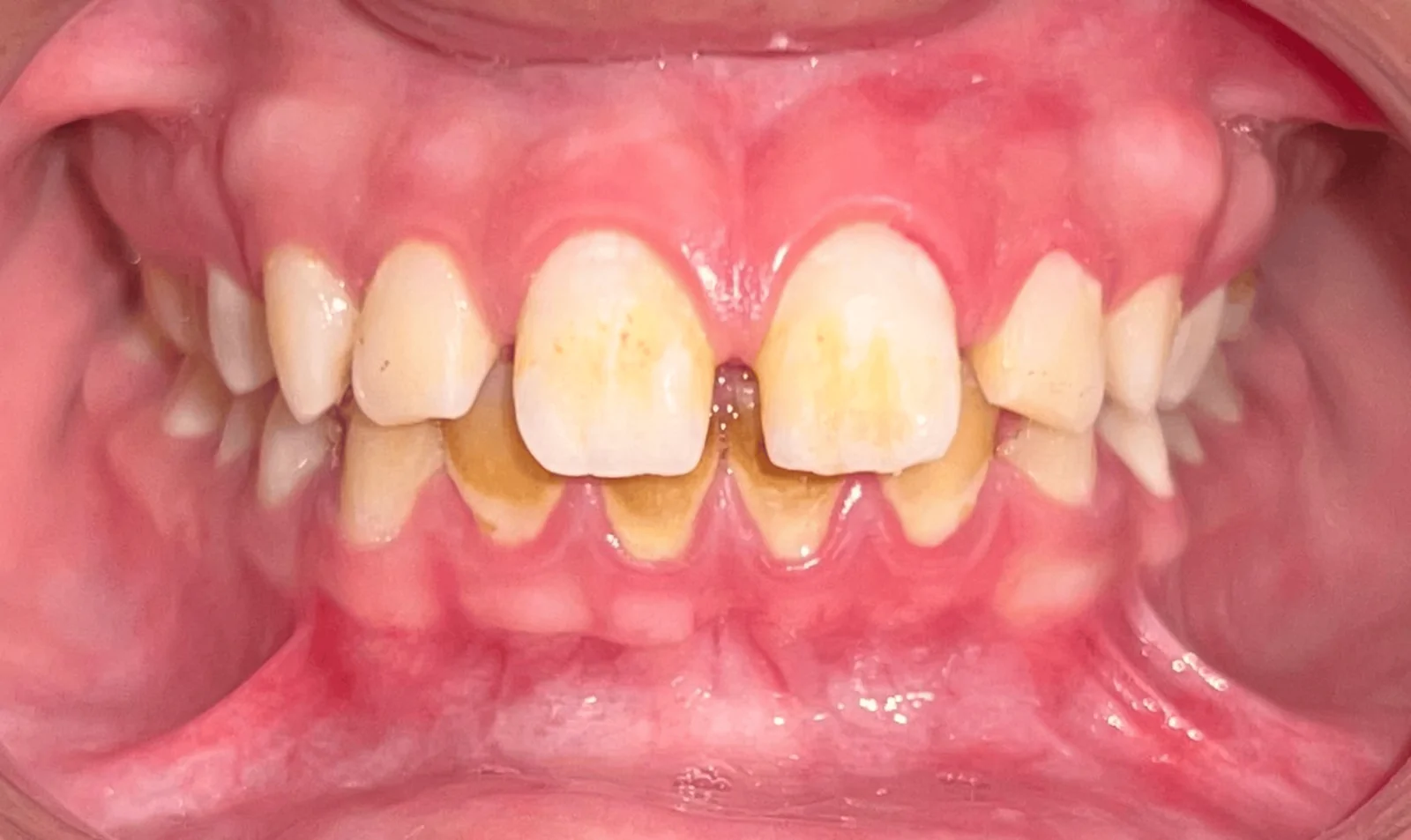
Intraoral view showing dental calculus accumulation on the maxillary and mandibular anterior teeth.

**Figure 4:**
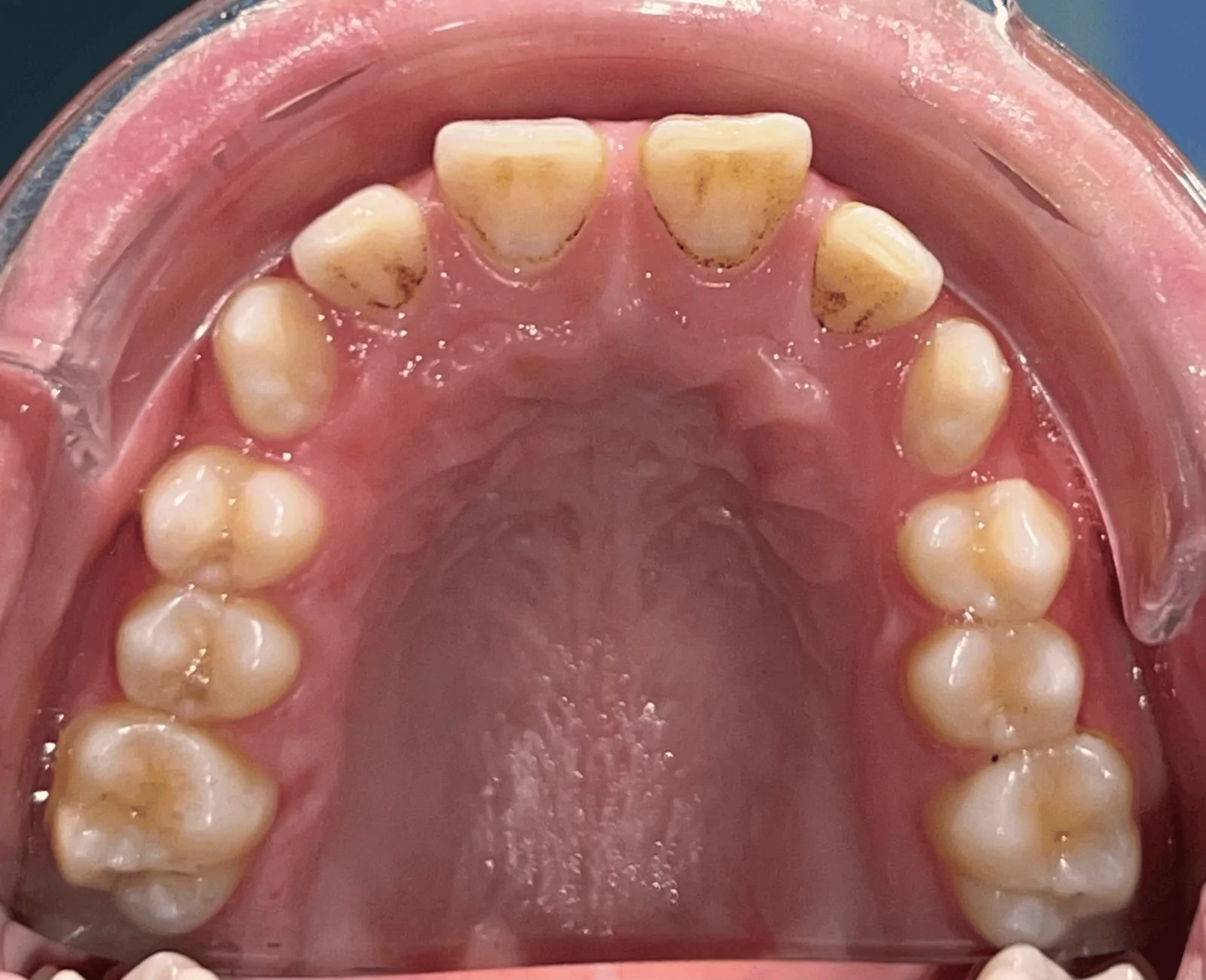
Intraoral view showing dental calculus accumulation on the maxillary anterior teeth.

**Figure 5:**
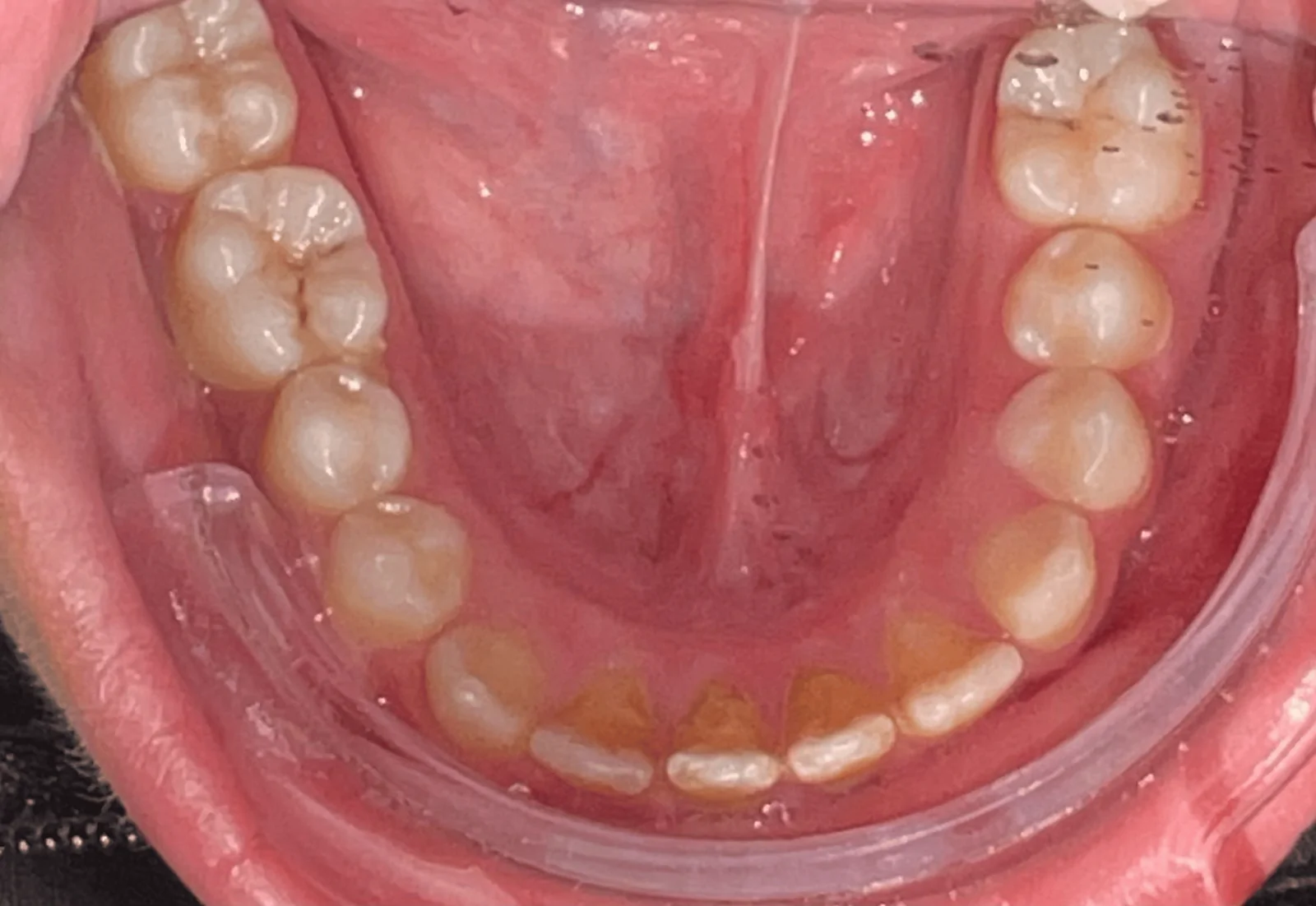
Intraoral view showing dental calculus accumulation on the mandibular anterior teeth.

At the initial dental examination, extraoral examination revealed no facial asymmetry, cutaneous lesions, or cervical lymphadenopathy. A 5-mm aphthous ulcer was noted on the external surface of the upper lip (Figure 1). Intraoral examination revealed poor oral hygiene and a large aphthous ulcer measuring approximately 8 mm in diameter on the left lateral border of the tongue (Figure 2), along with significant calculus accumulation in the maxillary and mandibular anterior regions and generalized gingival inflammation (Figures 3-5). The patient also experienced difficulty maintaining adequate oral hygiene, primarily because of pain caused by the ulcerative lesions.

**Figure 6:**
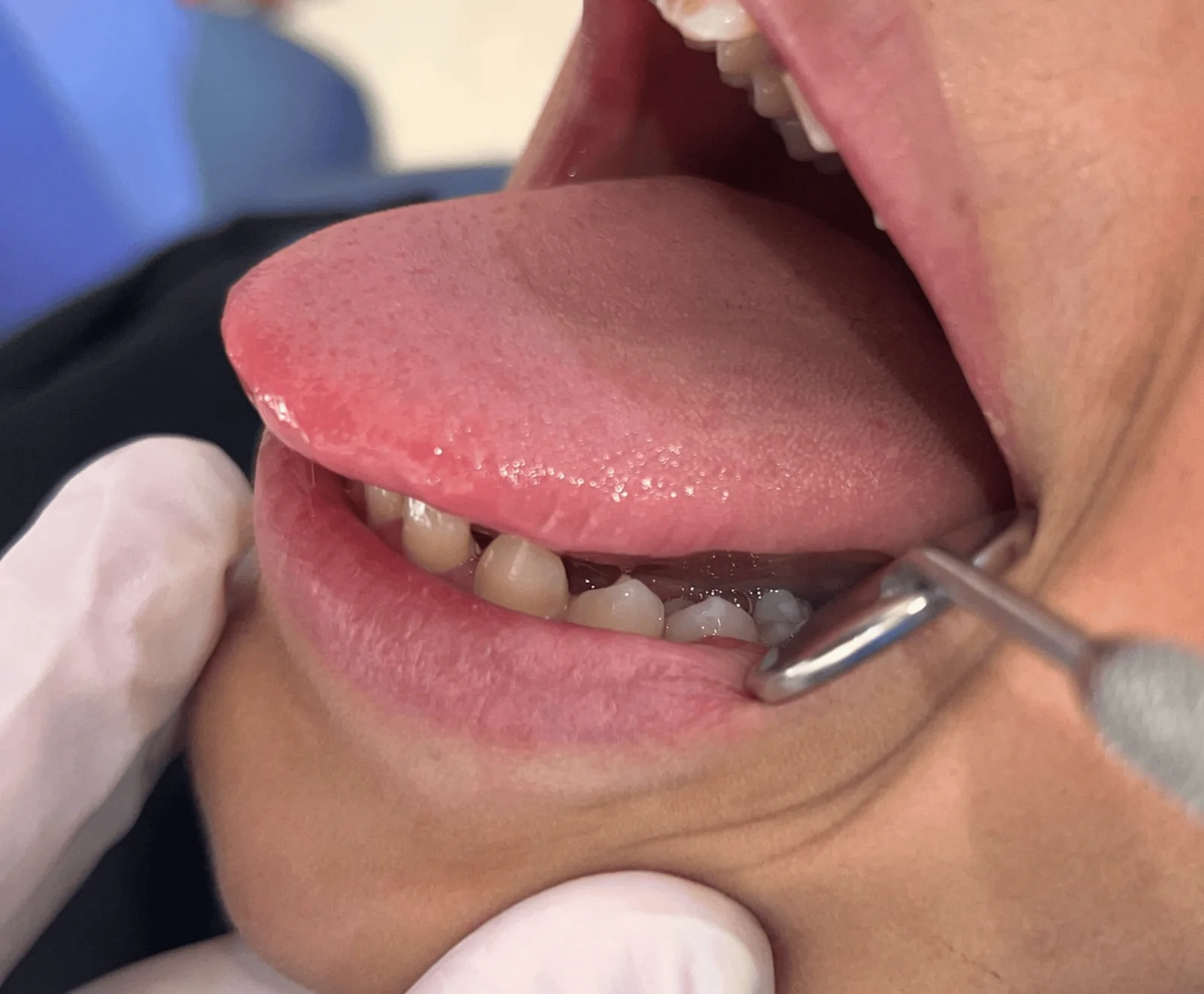
ntraoral view at the 10-day follow-up showing healing of the previously observed aphthous ulcer on the left lateral border of the tongue.

**Figure 7:**
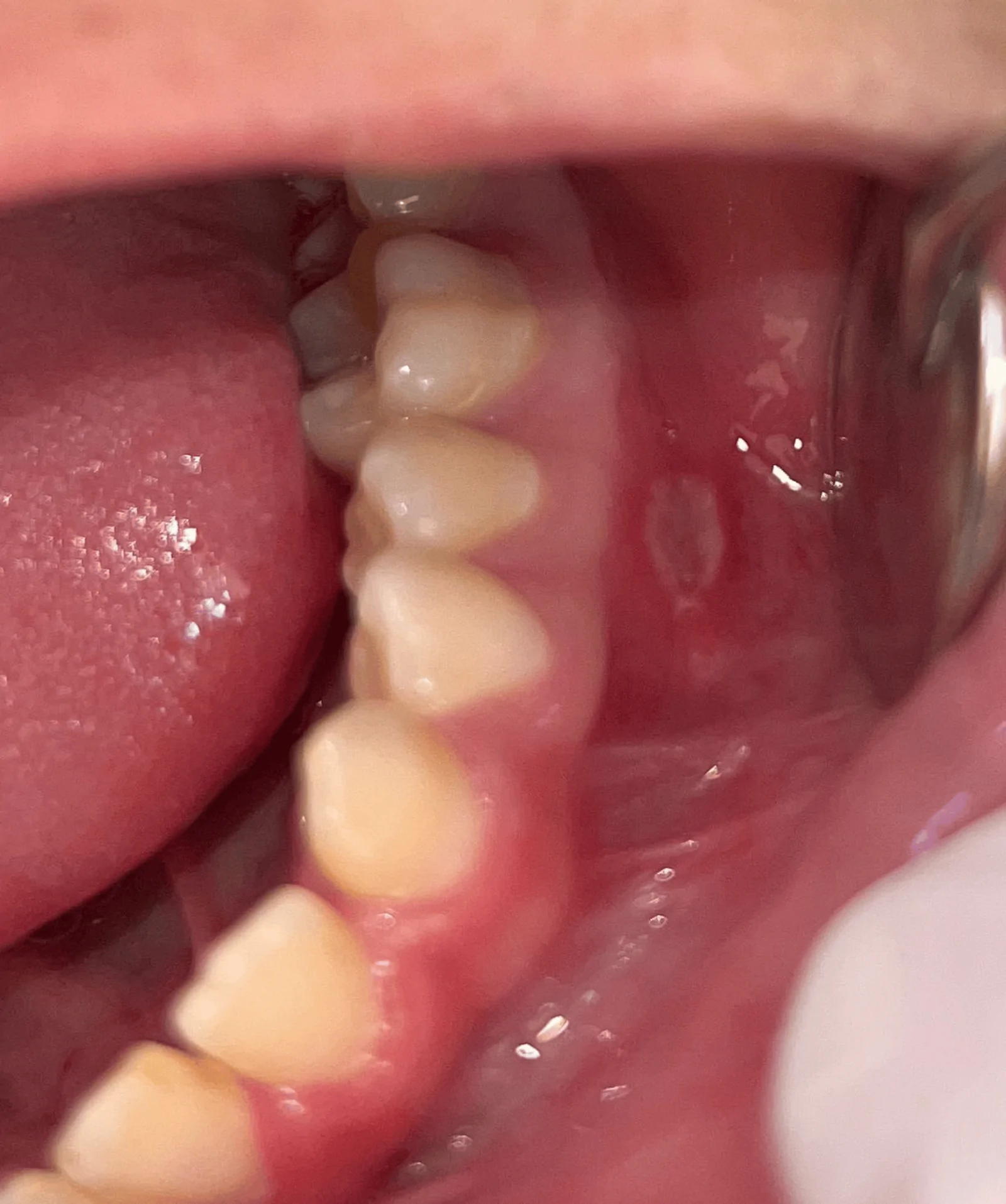
Intraoral view showing a new aphthous ulcer in the vestibular sulcus adjacent to the mandibular left first and second premolars (teeth 34 and 35).

At the 10-day follow-up visit, healing of the previously noted lesions was observed (Figure 6), alongside the emergence of new aphthous ulcers, particularly on the inner aspect of the upper lip and in the vestibular sulcus opposite teeth 34 and 35 (Figure 7).

**Figure 8:**
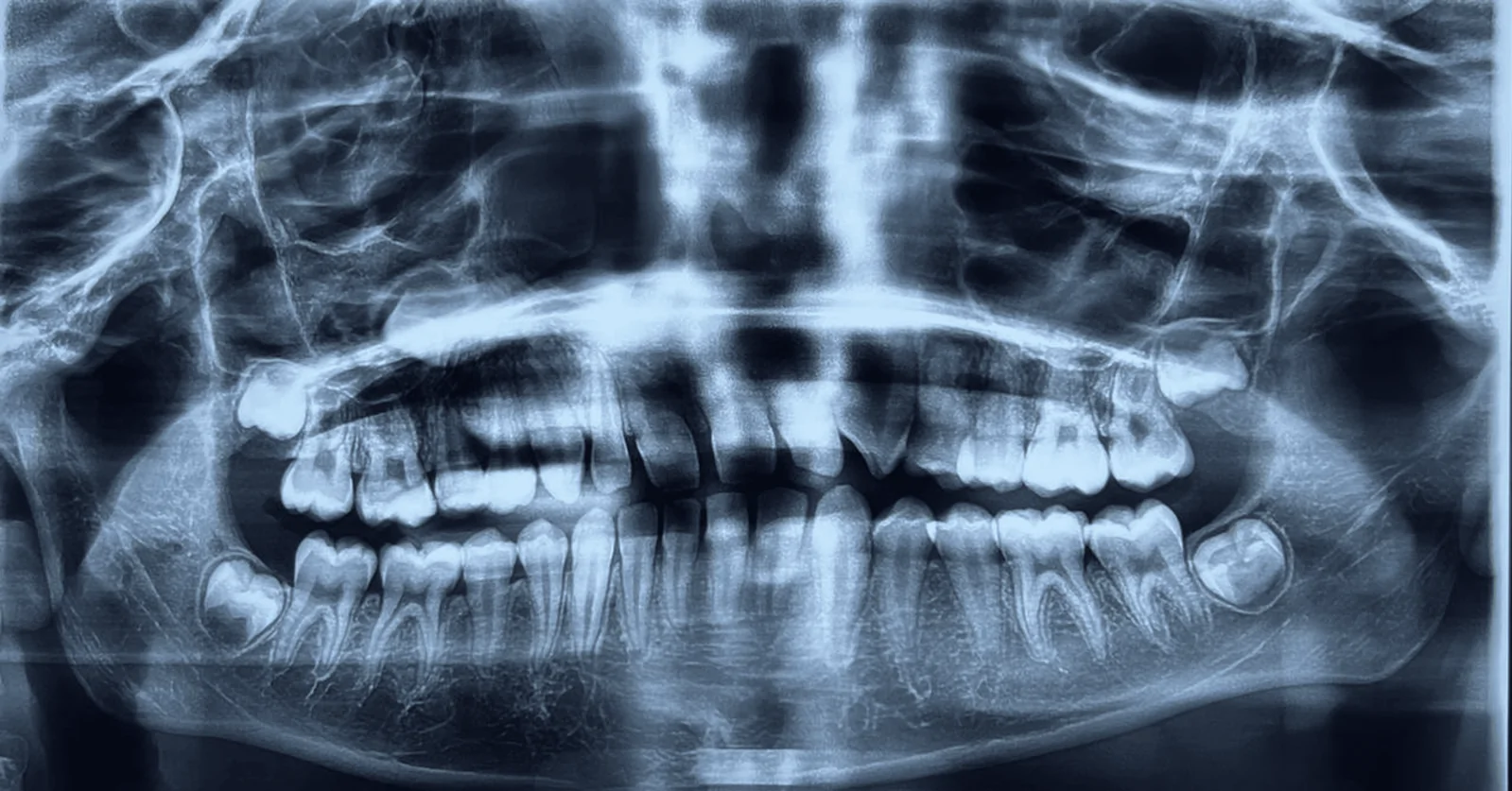
Panoramic radiograph of the patient.

A panoramic radiograph was obtained as part of the comprehensive dental assessment to evaluate the dental status and investigate potential hard-tissue involvement related to BS. Radiographic examination revealed no evidence of cortical bone alterations, mandibular lesions, or temporomandibular joint abnormalities (Figure 8).

Laboratory investigations showed a normal blood count and the presence of the HLA-B*51/B5 allele (Table 2).

**Table 2:** Laboratory investigations performed at the time of diagnosis. HLA: Human leukocyte antigen; MCH: Mean corpuscular hemoglobin; MCHC: Mean corpuscular hemoglobin concentration; MCV: Mean corpuscular volume; MPV: Mean platelet volume; RDW: Red cell distribution width.

Parameter	Patient value	Reference range
WBC	3.66 × 10⁹/L	4.00-10.00 × 10⁹/L
Neutrophils	1.72 × 10⁹/L	2.00-7.50 × 10⁹/L
Lymphocytes	1.80 × 10⁹/L	0.80-4.00 × 10⁹/L
Monocytes	0.37 × 10⁹/L	0.12-1.20 × 10⁹/L
Eosinophils	0.05 × 10⁹/L	0.02-1.00 × 10⁹/L
Basophils	0.02 × 10⁹/L	0.00-0.10 × 10⁹/L
RBC	4.88 × 10¹²/L	3.50-5.50 × 10¹²/L
Hemoglobin	13.7 g/dL	11.0-17.0 g/dL
Hematocrit	42.40%	35.0-50.0%
Mean corpuscular volume (MCV)	87.3 fL	80.0-100.0 fL
Mean corpuscular hemoglobin (MCH)	28.1 pg	27.0-34.0 pg
Mean corpuscular hemoglobin concentration (MCHC)	32.5 g/dL	30.0-37.0 g/dL
Red cell distribution width (RDW)	11.50%	11.0-16.0%
Platelets	247 × 10⁹/L	150-450 × 10⁹/L
Mean platelet volume (MPV)	9.7 fL	6.5-12.0 fL
ESR	8 mm/h	0-15 mm/h
CRP	0.01 mg/L	0.00-6.00 mg/L
HLA-B*51	Positive	Negative

Laboratory investigations were unremarkable except for mild leukopenia and neutropenia. The patient remained clinically asymptomatic, with no history of recurrent infections or signs suggestive of immunodeficiency. Inflammatory markers were within normal ranges at the time of evaluation.

Oral management

The management approach included an assessment of dietary habits, which revealed frequent snacking. Individualized, age-appropriate dietary counseling was provided to reduce between-meal food intake and improve oral health-related behaviors, with the aim of enhancing oral health-related quality of life and preventing the progression of mucosal lesions.

**Figure 9:**
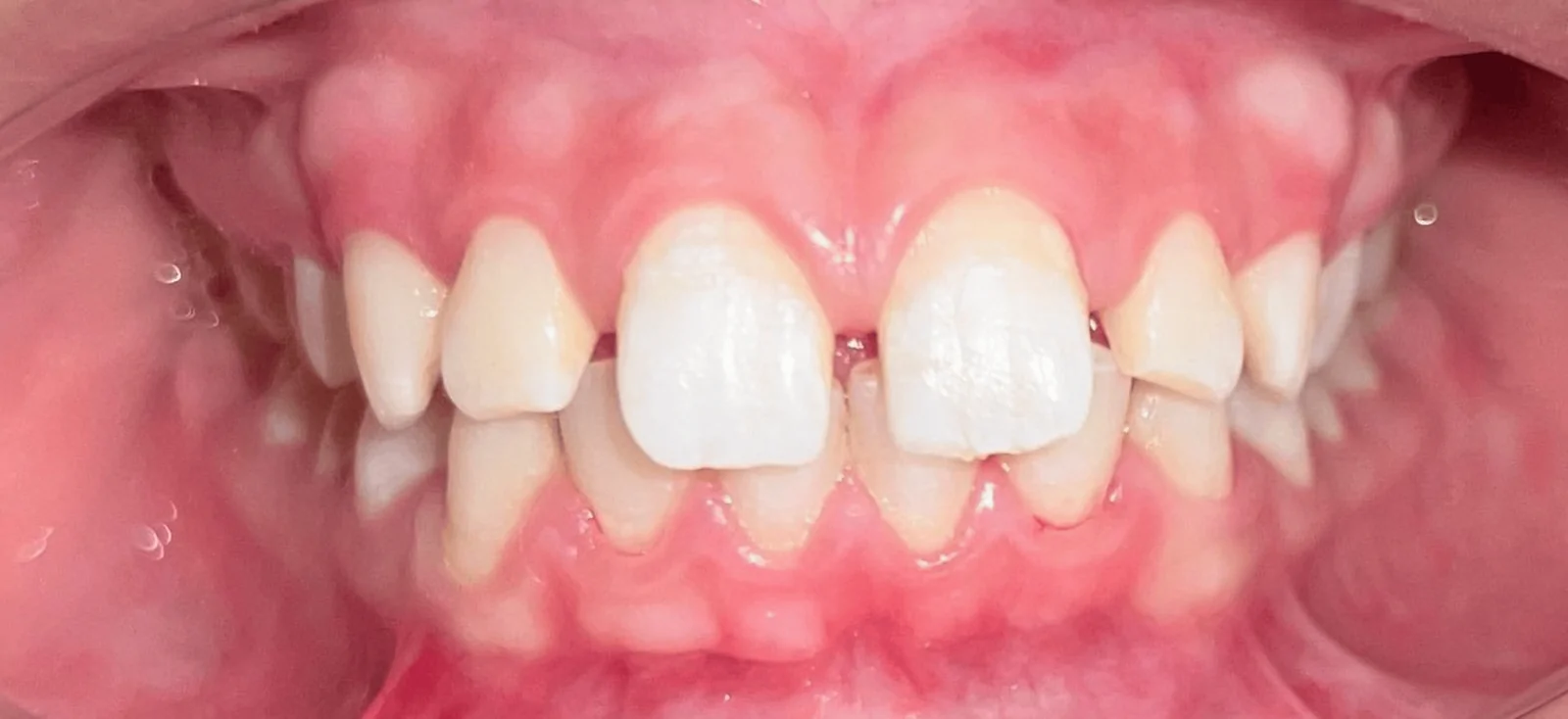
Full-mouth scaling, oral hygiene instructions, and topical treatment with propolis and corticosteroids led to clinical improvement and stabilization of aphthous ulcers at follow-up

**Figure 10:**
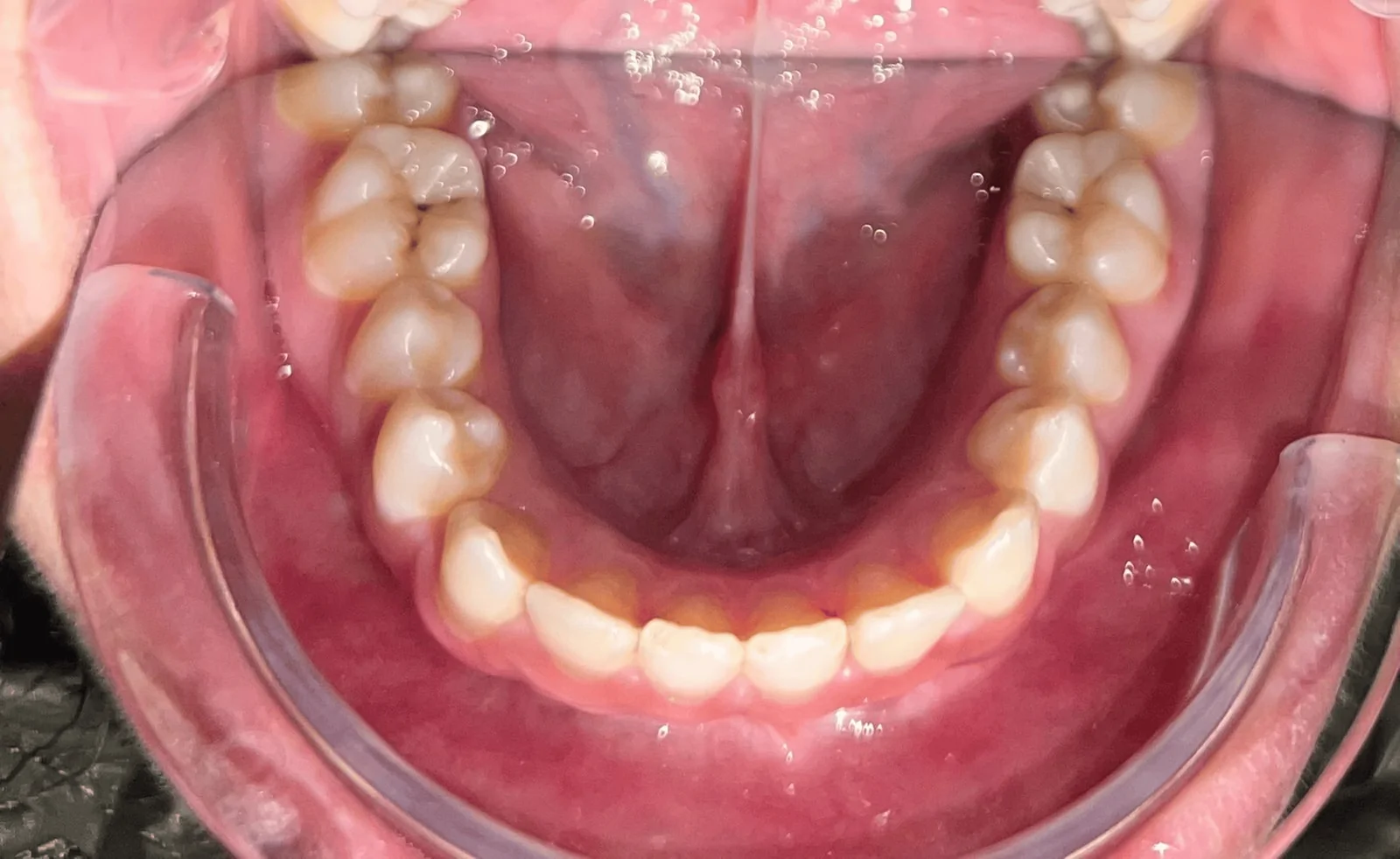
Mandibular view showing improved gingival contour and plaque control.

**Figure 11:**
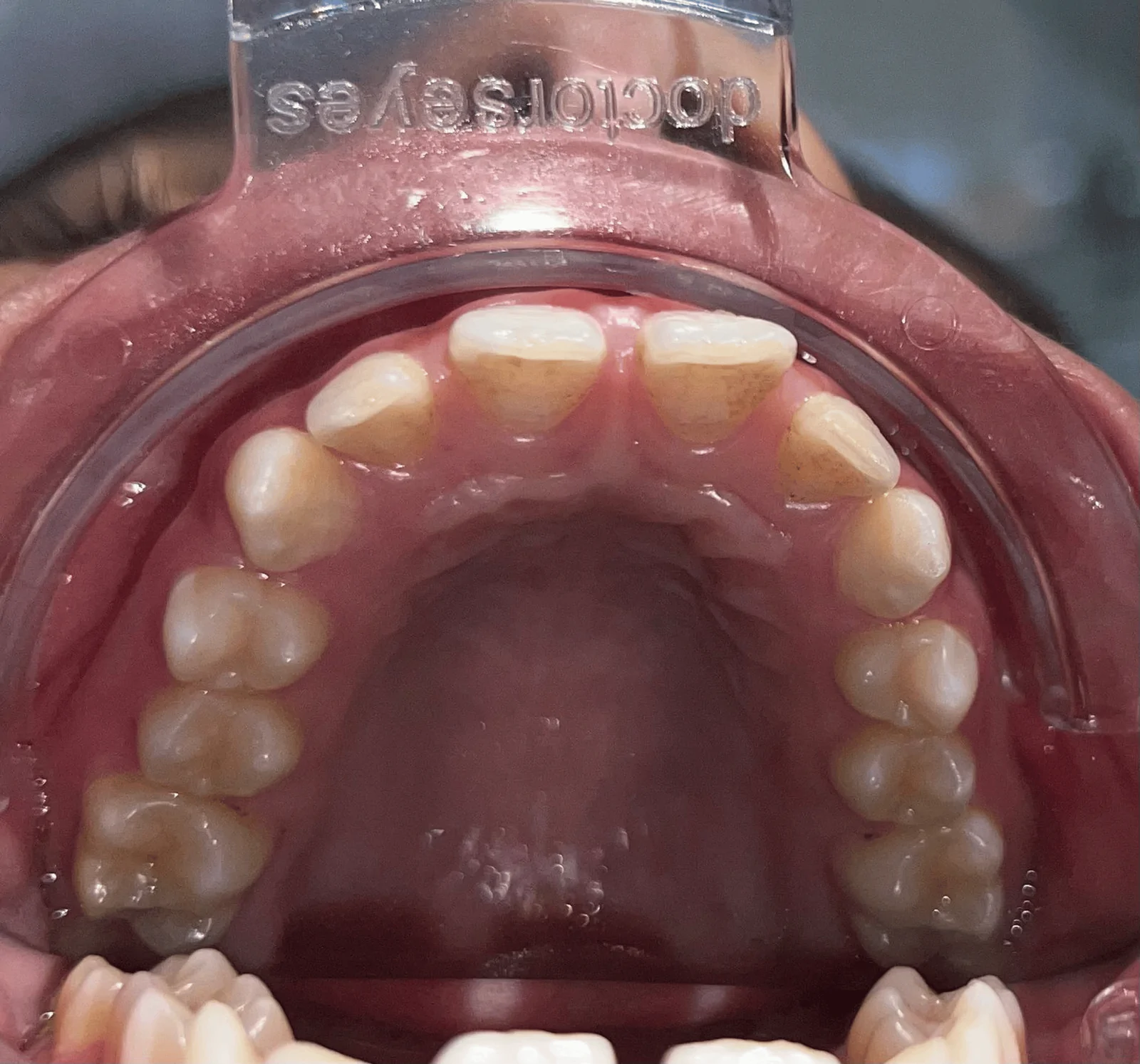
Maxillary view demonstrating reduced gingival inflammation.

Full-mouth scaling was performed to reduce the bacterial load and control gingival inflammation (Figures 9-11). This was combined with reinforcement of oral hygiene measures and symptomatic local management of oral ulcerations using topical corticosteroids and a propolis-based oral gel. These topical treatments were used during active episodes to promote healing and relieve discomfort.

Following the 10-day follow-up visit, the patient was followed for six months, with regular visits every 3-4 weeks to monitor clinical stability, assess the recurrence of oral lesions, and reinforce oral hygiene and dietary recommendations.

Written informed consent for the publication of this case report and the accompanying clinical images was obtained from the patient’s legal guardian (his mother).

At the 6-month follow-up, the patient had turned 12 years old and remained under regular multidisciplinary care. Although recurrent oral ulcerations continued to occur, consistent with the relapsing nature of BS, symptom severity was controlled through topical treatment, reinforcement of oral hygiene measures, and regular dental monitoring, resulting in satisfactory maintenance of the patient’s oral health.

## Discussion

BS most commonly begins in young adulthood, typically during the third decade of life, with a slight male predominance. Nevertheless, pediatric-onset disease is well documented. Onset before the age of 16 has been reported in approximately 4% to 26% of cases, with a mean age at onset ranging from 8 to 12 years [4]. In rare cases, Behçet-like manifestations may occur even earlier, including during the neonatal period, most often in infants born to mothers with active disease during pregnancy [3,9]. Diagnosis in pediatric patients is frequently delayed, with an estimated mean delay ranging from three to five years [10]. This diagnostic pattern is broadly consistent with our observation, in which the patient’s mother reported the onset of recurrent oral ulcers associated with joint involvement as early as two years of age, whereas the definitive diagnosis was established only at the age of 8 years.

This diagnostic delay is mainly explained by the rarity of BS in the pediatric population, the nonspecific nature of recurrent oral aphthous ulcers, and the wide range of differential diagnoses that may initially be considered [2]. These findings highlight the importance of early recognition and multidisciplinary management to reduce the risk of severe systemic complications, particularly neurological and thrombotic involvement [4]. In addition, delayed diagnosis has been attributed to the limited sensitivity of currently available international classification criteria, which were primarily developed for adult populations and remain insufficiently validated in children, as well as to the scarcity of large-scale case-control studies [10,11].

In the present case, the diagnostic delay may be attributed to the very early onset of symptoms, with recurrent oral ulcers appearing at the age of two years, when such manifestations are often nonspecific and may not immediately raise suspicion of BS. As additional clinical features emerged, the diagnosis was subsequently established by the patient’s treating physician based on the overall clinical presentation. The clinical presentation was consistent with the 2014 ICBD, supporting the classification of this case as pediatric BS.

The association of the HLA-B51 allele with BS, together with the occurrence of familial clustering, supports a genetic predisposition to the disease. A positive family history has been reported in approximately 9% to 30% of pediatric cases, representing an important consideration during clinical assessment [3,12]. In our patient, HLA-B51 positivity was consistent with previous reports, although no family history of Behçet’s disease was identified. Beyond HLA-B*51, recent studies have identified TNFAIP3 mutations causing A20 haploinsufficiency (HA20) as a monogenic Behçet-like condition, particularly in children. Genetic testing may therefore be considered in selected patients with early-onset, familial, or atypical presentations but was not performed in our case [13]. The patient’s mother did, however, report a family history of psoriasis. Interestingly, several studies have described the occurrence of other immune-mediated diseases, including psoriasis, within the families of patients with BS, suggesting a shared immunogenetic background [5,8]. However, to date, no evidence supports a direct causal relationship between a family history of psoriasis and the development of BS. Both conditions are considered distinct entities with different primary genetic determinants, despite potential overlap within a broader spectrum of immune-mediated inflammatory diseases [8].

BS is a rare systemic vasculitis in children, and its diagnosis remains complex and is based primarily on clinical criteria. Its clinical presentation is highly heterogeneous, reflecting the involvement of blood vessels and multiple organ systems throughout the body [4]. Although a wide spectrum of clinical signs and manifestations may be observed during evaluation, nonspecific intraoral ulcerations play a predominant role and are considered key features in commonly used classification criteria [11].

Oral aphthous ulcers are the earliest and most frequent manifestation of Behçet’s disease (BD), occurring in approximately 96-100% of pediatric cases, often representing the initial clinical feature and preceding other mucocutaneous or systemic manifestations [2]. In contrast, ocular, cutaneous, gastrointestinal, neurological, vascular, and articular involvement occurs less frequently, underscoring the importance of recurrent oral ulcers as an early diagnostic clue and highlighting the pivotal role of dental practitioners in the early recognition of juvenile BS [2]. Recurrent oral ulceration may persist over extended periods, even during phases of remission of more severe systemic manifestations. Consequently, recurrent oral lesions can serve as useful clinical markers for monitoring disease activity and assessing therapeutic response [2,7]. Oral aphthous ulcers associated with Behçet’s disease are typically painful, non-scarring, and round. They are often small (less than 10 mm in diameter), superficial or deep, and have a yellowish fibrinopurulent base and a well-defined erythematous halo [1,7]. They preferentially occur on the tongue and buccal or oropharyngeal mucosa. They may appear spontaneously several years before the diagnosis is established or be triggered by minor trauma, including dental procedures, consistent with the pathergy phenomenon [1, 2].

In our patient, the lesions consisted of a 5-mm aphthous ulcer on the external surface of the upper lip and an 8-mm aphthous ulcer on the left lateral border of the tongue.

These oral lesions may occur in isolation or in association with genital involvement and may form part of a broader systemic presentation that includes cutaneous, ocular, articular, gastrointestinal, and neurological manifestations [4,11]. Compared with adults, PEDBD may present with a distinct clinical profile [11,14]. Musculoskeletal involvement is among the most common systemic manifestations and typically presents as non-erosive monoarthritis or oligoarthritis, predominantly affecting the large joints [4]. Similarly, Makmur et al. [14] described a higher frequency of articular and gastrointestinal involvement in pediatric patients, in contrast to adults, in whom ocular, cutaneous, and vascular manifestations are more prevalent.

In the present case, the clinical findings were consistent with the reported pediatric phenotype, characterized by recurrent oral ulcerations, a previous episode of genital ulceration, and articular involvement manifested as lower-limb arthralgia, without ocular, neurological, or gastrointestinal manifestations.

Several studies have suggested that the chronic inflammatory process in Behçet’s disease may adversely affect bone metabolism, potentially leading to reduced bone mineral density and an increased risk of osteoporosis [15, 16]. More recently, mandibular bone alterations, including decreased trabecular bone density, cortical bone resorption, and degenerative changes in the temporomandibular joint, have also been reported in patients with Behçet’s disease [16]. However, panoramic radiography in our patient did not reveal any cortical or temporomandibular joint abnormalities.

In pediatric patients, the diagnosis of Behçet’s disease remains challenging due to the heterogeneity of clinical manifestations and their often progressive nature [4,11]. Several conditions may mimic its presentation, including infectious diseases such as herpes simplex virus infection, syphilis, and HIV; nutritional deficiencies; cyclic neutropenia; autoinflammatory syndromes, including PFAPA syndrome and hyper-IgD syndrome; systemic lupus erythematosus; and chronic inflammatory bowel diseases [9, 11]. Multiple oral ulcers, morphological variability, diffuse erythematous borders, and involvement of the soft palate or oropharynx are considered suggestive clinical features of Behçet’s disease [2,7]. The diagnosis of BS remains primarily clinical and is supported by established classification criteria. In particular, the international pediatric classification criteria require the presence of at least three of six distinct categories: recurrent oral aphthosis, genital ulceration, skin involvement, ocular involvement, neurological signs, and vascular signs [2,9]. Despite the existence of well-established international classification criteria, Behçet’s disease has recently been described as a true “great masquerader” because of the marked heterogeneity of its clinical presentations and its relatively high rate of misdiagnosis [1].

Given the rarity and clinical heterogeneity of juvenile BS, we summarized the main clinical characteristics reported in the pediatric literature to provide a comprehensive overview of the disease spectrum and facilitate comparison with the present case (Table 3).

**Table 3:** Comparison of the oral and systemic manifestations of juvenile Behçet syndrome reported in the pediatric literature with those observed in the present case. HLA: Human leukocyte antigen; mg/day: Milligrams per day.

Study	Age group	Oral manifestations/findings	Systemic manifestations	Treatment
Piram M and Koné-Paut I [3]	Children (<16 years)	Recurrent oral aphthous ulcers (hallmark feature)	Genital ulcers, skin lesions, and ocular, articular, gastrointestinal, neurological, and vascular involvement	Colchicine, corticosteroids, and immunosuppressive therapy according to disease severity
Koné-Paut I [4]	Children (<16 years)	Recurrent oral aphthous ulcers	Genital ulcers, cutaneous lesions, uveitis, arthritis, and gastrointestinal and neurological involvement	Colchicine, corticosteroids, immunosuppressants, and biologic agents
Yıldız M et al. [11]	Children and adolescents (≤18 years)	Recurrent oral aphthous ulcers	Mucocutaneous, ocular, musculoskeletal, gastrointestinal, neurological, and vascular manifestations	Conventional and biologic therapies according to organ involvement
Alatyan MH et al. [9]	8-year-old Syrian girl	Deep, painful oral ulcer; recurrent oral ulcers; tongue scar	Chronic constipation, arthralgia, and HLA-B*51 positivity	Predo® mouthwash and paracetamol, followed by colchicine
Zabala-Sepúlveda LM et al. [10]	Patients aged <18 years	Recurrent oral ulcers (most common manifestation)	Ocular, cutaneous, musculoskeletal, gastrointestinal, neurological, and vascular manifestations	Conventional and biologic therapies
Present case	Male aged 11 years and 6 months at the initial dental examination	Recurrent oral aphthous ulcers, including an 8-mm ulcer on the lateral border of the tongue; gingival inflammation; poor oral hygiene	Previous genital ulceration and lower-limb arthralgia	Full-mouth scaling, oral hygiene instructions, topical corticosteroids, propolis-based oral gel, and colchicine (1 mg/day)

Children with Behçet’s disease have been reported to exhibit poorer oral hygiene than healthy controls, as reflected by higher plaque index values in previous studies [7,17]. This difference may be attributed to a reduced frequency of toothbrushing among affected patients [18]. Indeed, recurrent aphthous ulcerations are often painful and may compromise oral hygiene practices by making toothbrushing difficult because of ulcerative involvement of the oral mucosa [7,16,18]. In the present case, the patient exhibited poor oral hygiene, with significant dental calculus accumulation on the mandibular incisors, and reported irregular toothbrushing because of pain associated with recurrent oral ulcerations. These observations are consistent with previously reported findings.

Beyond their impact on oral hygiene, such alterations in the oral environment may contribute to the pathophysiological mechanisms of the disease [5,6]. Growing evidence suggests a potential role of the oral microbiota in the pathogenesis of BS [2,6]. The frequent occurrence of initial disease manifestations in the oral mucosa, together with mucocutaneous or systemic exacerbations reported after certain dental procedures, suggests a close interaction between oral microorganisms and the host immune response [2,6]. Furthermore, improvements in oral hygiene and the elimination of dental and periodontal infectious foci have been associated with a reduction in the frequency of aphthous recurrences, highlighting the potential role of oral health in disease modulation [2, 6]. In this context, the patient underwent comprehensive oral management, including oral hygiene instruction and motivation, periodontal therapy, and elimination of local inflammatory factors. The clinical improvement observed after these interventions is consistent with the available literature and supports the relevance of preventive strategies aimed at reducing the oral bacterial load and local inflammation.

Accordingly, a widely proposed hypothesis is that BS results from an aberrant immune-inflammatory response directed against microbial antigens in genetically predisposed individuals [6,12]. Among the microorganisms implicated, oral streptococci, particularly *Streptococcus sanguinis*, have been proposed to play a central role in the initiation, persistence, and exacerbation of the disease [2,5,6]. This hypothesis is supported by several lines of evidence, including the exacerbation of symptoms following dental procedures or intradermal injection of streptococcal antigens, the increased prevalence of atypical streptococcal strains within the oral microbiota of patients with BS, and the clinical improvement reported with antistreptococcal therapy [2, 6]. In addition, certain strains of *Streptococcus sanguinis* have been shown to induce the production of proinflammatory cytokines by epithelial cells and may trigger aberrant immune responses through molecular mimicry between microbial antigens and host proteins. Collectively, these findings support the hypothesis that oral microorganisms, particularly streptococci, may contribute to the initiation and perpetuation of the inflammatory processes characteristic of BS [5, 6,12].

The management of BS depends on the site and severity of the clinical manifestations. In the absence of widely established pediatric-specific treatment guidelines, therapeutic strategies are largely extrapolated from adult data, particularly the European League Against Rheumatism (EULAR) recommendations [4,11,19]. Topical corticosteroids, such as triamcinolone acetonide, are considered first-line therapy for isolated oral aphthous ulcers and genital lesions, whereas topical sucralfate may be used as an alternative or in combination to enhance symptomatic control [19]. Colchicine is considered a first-line systemic therapy for preventing recurrent mucocutaneous manifestations [4,19]. Systemic corticosteroids are reserved for refractory or severe disease, either as monotherapy or in combination with immunosuppressive agents, particularly in cases of cutaneous or mucocutaneous involvement resistant to local therapies or colchicine [19]. Disease-modifying antirheumatic drugs (DMARDs), including azathioprine, methotrexate, mycophenolate mofetil, and sulfasalazine, may be used as second-line treatments in selected cases, depending on the extent of organ and systemic involvement [4, 19].

In the present case, the patient was treated with colchicine at a dose of 1 mg/day for manifestations predominantly characterized by recurrent oral mucosal involvement associated with articular symptoms. Oral ulcers were managed locally using topical corticosteroids and propolis as adjunctive care for symptom relief. A propolis-based gel was preferred over the spray formulation because spray application was reported to cause discomfort when applied directly to ulcerated lesions. Propolis was used based on its reported anti-inflammatory and wound-healing properties in recurrent aphthous stomatitis, although evidence supporting its efficacy in BS remains limited [20]. During follow-up, improved patient comfort and progressive healing of the oral ulcers were observed. Nevertheless, the specific contribution of propolis cannot be determined because of the concomitant colchicine therapy and the natural course of ulcer healing. Regular follow-up was maintained to monitor the clinical course, identify possible recurrences, and adapt supportive management according to the patient’s needs. This approach highlights the importance of integrating oral care into the multidisciplinary management of pediatric BS and contributes to maintaining oral health and providing comprehensive dental care [2,19].

## Conclusions

This case highlights that recurrent oral lesions may represent an early manifestation of pediatric BS and should prompt clinicians to consider this diagnosis when accompanied by suggestive systemic features. It emphasizes the role of pediatric dentists in recognizing oral manifestations and contributing to multidisciplinary care aimed at managing oral symptoms, preserving oral health, and potentially improving patients’ quality of life. In this child, dental follow-up, combined with ongoing colchicine therapy and local measures, including topical corticosteroids and propolis-based care, allowed regular monitoring and symptomatic management of oral lesions. Although recurrent ulcers continued to develop, healing of the observed lesions and satisfactory maintenance of oral health were achieved during follow-up. This observation contributes to the existing knowledge on the dental management of pediatric BS while highlighting the need for further studies to better characterize oral involvement and define the role of dental care in the comprehensive management of affected children.
